# Impact of Direct Contact and Ingestion of Selected Insecticides on the Predator *Harmonia axyridis* of Citrus Psyllids

**DOI:** 10.3390/insects16020126

**Published:** 2025-01-27

**Authors:** Jing Pan, Gaoqi Cheng, Minjue Liu, Xiangfeng Pan, Zhigang Ouyang, Zhanjun Lu, Yimin Du

**Affiliations:** 1 College of Life Sciences, Gannan Normal University, Ganzhou 341000, China; pan215662131@163.com (J.P.);; 2 National Navel Orange Engineering Research Center, Ganzhou 341000, China; 3 Jiangxi Provincial Key Laboratory of Pest and Disease Control of Featured Horticultural Plants, Ganzhou 341000, China

**Keywords:** toxicity, non-target organism, coccinellid, *Diaphorina citri*, IPM, lambda-cyhalothrin, thiamethoxam

## Abstract

The Asian citrus psyllid, *Diaphorina citri*, serves as the primary vector for Huanglongbing (HLB) by transmitting *Candidatus* Liberibacter asiaticus. Lambda-cyhalothrin and thiamethoxam are commonly employed for the control of *D. citri*. The multicolored Asian lady beetle, or harlequin ladybird, *Harmonia axyridis*, is an important predator of *D. citri* in both greenhouse and field settings. The effectiveness of integrated pest management (IPM) relies on the judicious use of selective insecticides that minimize harm to natural enemies. However, the effects of these insecticides on *H. axyridis*’ predation of *D. citri* remain thoroughly unexplored. In this study, we assessed the toxicity of lambda-cyhalothrin and thiamethoxam to *H. axyridis* and examined their impact on the functional response of this predator to *D. citri* using direct contact exposure methods. We also evaluated the indirect effects on predator voracity through ingestion exposure. Our results demonstrated that exposure to both insecticides at LC_50_ concentrations significantly prolonged the developmental durations of *H. axyridis* larval stages. The type-II functional response model effectively described the prey consumption patterns of *H. axyridis*, revealing a significant reduction in predation capacity across all life stages, particularly among second instar larvae, which experienced reductions of 85.30% and 88.58% following lambda-cyhalothrin and thiamethoxam treatments, respectively. Furthermore, *H. axyridis*’ predation significantly declined when feeding on *D. citri* contaminated at LC_50_ concentrations. These findings indicate that lambda-cyhalothrin and thiamethoxam adversely affect the predation of *H. axyridis*, both via direct contact and ingestion. Evaluating the potential impacts of these insecticides on *H. axyridis* is critical for the development of effective IPM strategies targeting *D. citri*.

## Simple Summary

*Diaphorina citri* is a significant pest that acts as a vector for bacteria belonging to the genus *Candidatus* Liberibacter, which are suspected agents of Huanglongbing (HLB) disease in citrus. *Harmonia axyridis* Pallas, 1773 (Coleoptera: Coccinellidae) is an important natural enemy in the control of *D. citri*. This study assessed the impacts of the commonly used insecticides lambda-cyhalothrin and thiamethoxam on *H. axyridis*’ predation of *D. citri* through two exposure routes. First, the acute toxicity of these insecticides to both *H. axyridis* and *D. citri* was evaluated. The results indicated that the LC_50_ values for lambda-cyhalothrin and thiamethoxam against the second instar larva of *H. axyridis* were 0.870 mg/L and 20.861 mg/L, respectively. In the direct contact exposure bioassay, dipping the second instar larvae of *H. axyridis* in solutions of lambda-cyhalothrin and thiamethoxam at their LC_50_ concentrations significantly extended the developmental durations of both second and third instar larval stages. Furthermore, the functional response indicated a marked reduction in the predation capacity of *H. axyridis* across all developmental stages. In the ingestion exposure bioassay, adult *D. citri* contaminated with the LC_50_ of each insecticide exhibited a significant decline in voracity, subsequently diminishing *H. axyridis*’ predation effectiveness. Our findings demonstrate that lambda-cyhalothrin and thiamethoxam adversely affect the development of *H. axyridis* and its predation on *D. citri*, a consideration that should be factored into biological control strategies targeting citrus psyllids.

## 1. Introduction

The Asian citrus psyllid, *Diaphorina citri* Kuwayama (Hemiptera: Liviidae), is a significant pest in the global citrus industry, known for its direct feeding on plant sap, which leads to shoot tip wilting and branch deformities [1,2]. Of greater concern is its role in indirectly causing damage by transmitting *Candidatus* Liberibacter asiaticus (CLas), a phloem-restricted bacterium responsible for Huanglongbing (HLB), the most devastating citrus disease [3]. Trees infected with HLB exhibit a complex array of symptoms, including chlorotic leaves and misshapen and small fruit [4,5]. Despite extensive research on HLB over the years, no effective cure currently exists for the disease. Consequently, preventing and controlling *D. citri* has emerged as the primary strategy for managing HLB.

Control of *D. citri* primarily relies on insecticides, such as pyrethroids and neonicotinoids, which are broad-spectrum agents targeting various sap-sucking pests [6,7,8]. While the application of these chemical agents can swiftly diminish psyllid populations, their widespread use has resulted in the emergence of insecticide resistance [9,10]. To mitigate reliance on chemical controls, the implementation of integrated pest management (IPM) strategies, including biological control methods, is essential for the management of psyllids [3,11]. Natural enemies, both predatory and parasitic, play a crucial role in the biological control of *D. citri*, encompassing various ladybirds, lacewings, and parasitoids [12,13,14,15]. *Tamarixia radiata* (Waterston) is recognized as the dominant parasitoid of *D. citri*, achieving a parasitism rate of approximately 36–46% in China [16]. However, Guo et al. unexpectedly observed that *T. radiata* nymphs can acquire CLas, which the subsequent adult parasitoids transmit to *D. citri* nymphs [17]. In contrast to *T. radiata*, which limits its reproductive capacity by solely parasitizing *D. citri*, the euryphagic harlequin ladybird, *Harmonia axyridis*, preys on various small insect pests [18,19]. Originally native to Asia, *H. axyridis* has been introduced to Europe as a biological control agent against aphids and coccids [20], and it can now be mass-produced conveniently in biocontrol factories for field application. Both larval and adult stages of *H. axyridis* predominantly consume the thoracic and abdominal regions of adult *D. citri*, exhibiting predation rates consistent with a type II Holling’s functional response across varying *D. citri* densities [13].

A fundamental aspect of integrated pest management (IPM) strategies is the combination of insecticides with biological control methods [21]. Natural enemies are inevitably exposed to spray residues or droplets while foraging for prey or hosts; thus, it is crucial to identify insecticides that pose minimal harm to these beneficial organisms [22,23]. Numerous studies have demonstrated that insecticides can have lethal effects on natural enemies, with mortality being the most direct consequence [24]. Additionally, sublethal effects, which do not result in immediate mortality but adversely impact the physiology and behavior of beneficial arthropods, can also be significant [21].

Lambda-cyhalothrin and thiamethoxam are insecticides widely employed against psyllids in Ganzhou citrus orchards [14]. Multiple studies indicate their detrimental effects on natural enemies, particularly *H. axyridis* [23,25,26]. Lambda-cyhalothrin exhibits relatively high acute toxicity to both larvae and adults of *H. axyridis*, with nearly all instars succumbing within three hours of ingesting lambda-cyhalothrin-treated aphids [25,27]. Thiamethoxam also has transgenerational impacts on *H. axyridis*, reducing larval and pupal survival in the F1 generation when applied to cotton seeds [28]. Moreover, sublethal effects of thiamethoxam diminish the population growth of *H. axyridis* via both contact and leaf-dip applications, thereby influencing the biological control of *Myzus persicae* [22]. The application of thiamethoxam at its LC_50_ significantly increases predator mortality, and surviving individuals exhibit a reduced predation rate [29].

While most research has concentrated on the sublethal effects of insecticides on the ontogeny, survival, and reproduction of *H. axyridis*, no prior study has assessed the potential impact of lambda-cyhalothrin and thiamethoxam on the predation capacity of *H. axyridis* against *D. citri*, despite the critical importance of predation ability for the effective field application of natural enemies. Therefore, this study investigates the lethal toxicity of lambda-cyhalothrin and thiamethoxam on *H. axyridis* and examines the functional response and voracity of *H. axyridis* toward *D. citri* through various exposure routes. The findings provide essential guidance for the rational application of lambda-cyhalothrin and thiamethoxam and may facilitate the development of IPM programs incorporating *H. axyridis* for managing citrus psyllids.

## 2. Materials and Methods

### 2.1. Biological Materials

Adults of *D. citri* and *H. axyridis* were collected from an orchard at the National Navel Orange Engineering Research Center in Ganzhou, China, during the spring of 2021. *D. citri* was reared on *Citrus sinensis* cv. Newhall in nylon net insect rearing cages within a greenhouse maintained at 27–28 °C and 60–65% relative humidity under a 14:10 h light: dark photoperiod. The *H. axyridis* colony was sustained using both adult and nymph stages of *D. citri*. All bioassays were performed with the second laboratory generation of *H. axyridis*.

### 2.2. Insecticide Preparation

Thiamethoxam (purity 99%, Yangzhou AnaStandard Biotechnology Co., Ltd., Yangzhou, China) and lambda-cyhalothrin (purity 96%, Jiangsu Tianze Chemical Co., Ltd., Changzhou, China) were procured for this study. Acetone and Tween 80 were obtained from the Chinese Medicines Group Chemical Reagent Co., Ltd., Shanghai, China. To prepare a 1000 mg/L stock solution, 0.0521 g of lambda-cyhalothrin and 0.0505 g of thiamethoxam were dissolved in 3 mL of acetone and subsequently diluted to 50 mL with a 0.1% Tween 80 solution. The resulting stock solution was then further diluted to various concentrations using a 6% acetone solution containing 0.1% Tween 80.

### 2.3. Insecticides Toxicity on D. citri and H. axyridis

Toxicity assessments on *D. citri* were conducted using the leaf-dip method as outlined by Naeem et al. [30]. Five concentrations of each insecticide were prepared: lambda-cyhalothrin at 5, 10, 20, 40, and 80 mg/L and thiamethoxam at 0.5, 1, 1.5, 3, and 9 mg/L, each with five replicates. In each bioassay, Navel orange leaves were immersed in the respective insecticide solutions and allowed to air dry at room temperature for one hour. The petioles of the leaves were wrapped in sponges saturated with water to maintain freshness. Subsequently, 30 adult psyllids were introduced onto the treated leaves within transparent plastic Petri dishes (90 mm diameter, 10 mm height), which were sealed with Parafilm and placed in a climate chamber. Ventilation holes were created on the top surface using insect pins. A control treatment consisted of citrus leaves treated with a 6% acetone solution containing 0.1% Tween 80. The treated psyllids were maintained under experimental conditions as described in Section 2.1. Mortality was assessed after 24 h, with individuals that did not respond to prodding with a brush considered dead.

For *H. axyridis*, recognizing the potential for insecticides to directly contact the cuticle when ladybirds feed on psyllids in the orchard, toxicity tests were performed using the insect-dipping method [31]. Second instar larvae of *H. axyridis* were dipped in thiamethoxam solutions or 0.1% Tween 80 water for five seconds, then allowed to dry in a Petri dish. Each larva was subsequently transferred to a separate Petri dish, sealed with Parafilm (with ventilation holes), and provided with sufficient psyllids as food. Each group of 10 *H. axyridis* larvae constituted one replicate, with each treatment replicated five times. Mortality was recorded after 24 h following the same criteria as described above.

### 2.4. Sublethal Effect on Developmental Stage Traits of H. axyridis

To assess the sublethal effects of insecticides on *H. axyridis*, LC_50_ doses for lambda-cyhalothrin (0.870 mg/L) and thiamethoxam (2.030 mg/L) were utilized on second instar larvae. Following immersion in the LC_50_ solutions as detailed in Section 2.3, each treated larva was placed in an individual Petri dish covered with perforated Parafilm and provided with sufficient psyllids as food. Each insecticide treatment, along with the control, was replicated 20 times. The Petri dishes were maintained in climatic chambers and monitored until female *H. axyridis* adults laid eggs. The developmental duration, pupal period, and adult pre-oviposition period (APOP) of *H. axyridis* were subsequently recorded.

### 2.5. Sublethal Effect on Predatory Responses of H. axyridis

The predatory functional responses of *H. axyridis* to *D. citri* were assessed following the methodology outlined by Huang et al. [13]. Second instar larvae through to adult stages of *H. axyridis* were utilized for the functional response analysis. A layer of moist filter paper was placed at the base of 90 mm diameter, 10 mm high Petri dishes. A fresh citrus leaf was positioned on the moist filter paper, and second instar larvae of *H. axyridis*, previously soaked at LC_50_ doses of insecticides as described in Section 2.3, were introduced into each dish, with each treatment replicated five times. As the larvae developed, predation functional responses were recorded for each life stage. Preliminary observations indicated an increase in the number of psyllids consumed with advancing instar of *H. axyridis*. For second instar larvae, psyllid adult densities of 5, 10, 15, 20, and 25 were tested, while third instar larvae were provided with densities of 20, 30, 40, 50, and 60 psyllids. Both fourth instar larvae and adult *H. axyridis* were offered 50, 70, 90, 110, and 130 adult psyllids. The Petri dishes were then placed in an incubator, and the number of prey consumed in each replicate was recorded after 24 h. The daily predation rate was calculated, and Holling’s II functional response model was applied, expressed by the equation: *N*_a_ = *aN*_0_*T*/(1 + *aT*_h_*N*_0_), where *N*_a_ represents the number of prey consumed, *N*_0_ is the initial prey density, *T*_h_ is the handling time for *H. axyridis* preying on adult psyllids, *a* is the instantaneous attack rate, *T* is the total experimental duration (24 h), 1/*T*_h_ indicates the maximum daily predation rate, and *a*/*T*_h_ reflects the predation capacity.

### 2.6. Effect of Lambda-Cyhalothrin and Thiamethoxam on Predator Voracity

The voracity of *H. axyridis* in response to insecticide-contaminated *D. citri* was assessed by exposing the psyllids to LC_50_ concentrations of lambda-cyhalothrin (20.861 mg/L) and thiamethoxam (1.658 mg/L), as detailed in Section 2.3. Following a 24 h exposure, surviving psyllids were transferred to new Petri dishes (90 mm diameter, 10 mm high) with densities set at 25 for second instar larvae, 60 for third instar larvae, and 130 for both fourth instar larvae and adult *H. axyridis* per dish. This psyllid density was chosen to exceed the maximum daily consumption capacity of *H. axyridis* (refer to functional response results). Subsequently, starved larvae or adults of *H. axyridis* were individually introduced into the respective Petri dishes, and the number of psyllids consumed was recorded 24 h post-introduction. Each treatment was replicated three times.

### 2.7. Statistical Analysis

Data analysis was performed utilizing SPSS 20.0 software. LC_50_ values for lambda-cyhalothrin and thiamethoxam were determined through Probit analysis following a 24 h insecticide exposure [32]. Chi-square tests were employed to assess the goodness of fit for the virulence regression equation and the predatory response equation. The Shapiro–Wilk test assessed the normality of the distribution, while Levene’s test evaluated the homogeneity of variance. Both developmental periods and predator voracity exhibited a normal distribution. A one-way ANOVA was performed, followed by Tukey’s honestly significant difference (HSD) test for subsequent multiple comparisons. Results are presented as means ± standard error (S.E.).

## 3. Results

### 3.1. Insecticide Toxicity to H. axyridis and D. citri

The toxicity of lambda-cyhalothrin and thiamethoxam to second instar larvae of *H. axyridis* and adult *D. citri* is summarized in Table 1. The data conformed to the log-probit model, with no significant deviations from the regression equation detected. The median lethal concentrations (LC_50_) of lambda-cyhalothrin were found to be 0.870 mg/L for *H. axyridis* and 20.861 mg/L for *D. citri*. In contrast, the LC_50_ values for thiamethoxam were 2.030 mg/L for *H. axyridis* and 1.658 mg/L for *D. citri*. Notably, lambda-cyhalothrin demonstrated greater toxicity to *H. axyridis*, as indicated by a lower LC_50_ value compared to thiamethoxam.

### 3.2. Sublethal Effects on the Development of H. axyridis

**Figure 1:**
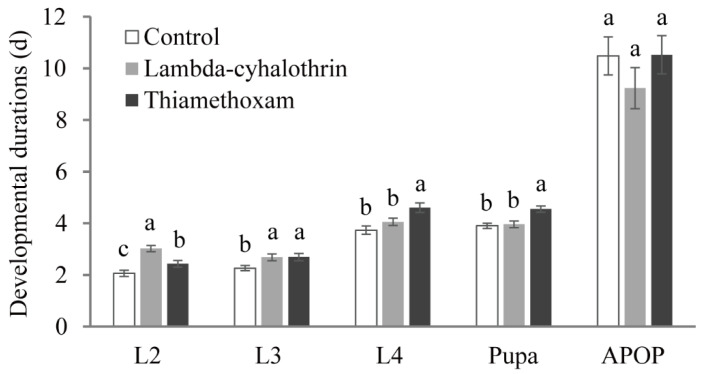
Toxic effects of two pesticide exposures on the development of *H. axyridis* during its immature stages. L2, second instar larva of *H. axyridis*; L3, third instar larva; L4, fourth instar larva; APOP, adult pre-oviposition period. Each histogram and error bar represented mean ± SE. Each treatment was replicated 20 times. Different lowercase letters within the same panel denoted significant differences among groups (*p* < 0.05).

Exposure to lambda-cyhalothrin and thiamethoxam solutions at LC_50_ concentrations, as detailed in Section 2.3, resulted in significantly prolonged developmental durations for both the second (*F*_(2, 54)_ = 51.605, *p* < 0.05) and third (*F*_(2, 52)_ = 7.319, *p* < 0.05) instar larval stages compared to the control (Figure 1). Specifically, lambda-cyhalothrin increased the duration by 0.96 days for second instar larvae and 0.42 days for third instar larvae. In contrast, thiamethoxam extended these durations by 0.37 days and 0.43 days, respectively. While lambda-cyhalothrin did not significantly affect the developmental durations of the fourth instar larvae and pupae, thiamethoxam significantly increased these periods by 0.87 days and 0.65 days, respectively. This suggests that thiamethoxam may exert a more prolonged effect on the development of *H. axyridis* compared to lambda-cyhalothrin. Additionally, the adult pre-oviposition period (APOP) did not differ significantly between the two insecticides.

### 3.3. Effects of LC_50_ Insecticides on the Predatory Function of H. axyridis

The functional response of *H. axyridis* to psyllid consumption over a 24 h period closely aligned with Holling’s disc model (type II) (Figure S1), as indicated by the Chi-square values in Table 2. In the untreated group, the predation capacity (*a*/*T_h_*) of ladybirds increased with developmental stage, peaking at 371.46 during the fourth instar larval stage before slightly declining to 349.79 in adults (Table 2). Subsequent to insecticide application, there was a decrease in the instantaneous attack rate (*a*) of second and third instar larvae of *H. axyridis*, with minimal alterations observed in fourth instar larvae and adults. The predation ability at all stages was markedly reduced after second instar larvae were exposed to insecticide solutions. In the case of lambda-cyhalothrin, the proportions decreased by 85.30%, 43.71%, 7.49%, and 6.53% from second instar larvae to adults compared to the untreated group, while thiamethoxam resulted in decreases of 88.58%, 43.71%, 6.21%, and 7.28%, respectively. These results indicate that both insecticides exerted substantial negative effects on the predation capacity of *H. axyridis*, with the most pronounced impact observed in second instar larvae, which diminished with the predator’s growth. Correspondingly, the handling time (*T_h_*) values also increased across all developmental stages following insecticide exposure.

### 3.4. Effect of Insecticide-Contaminated Psyllids on the Predation of H. axyridis

Exposure of *D. citri* to insecticides at LC_50_ significantly influenced the consumption number of psyllids by *H. axyridis* (Table 3). In second instar larvae, predation rates were notably reduced following thiamethoxam treatment (*F*_(2, 12)_ = 9.250, *p* < 0.05). However, no significant differences were observed in the third instar larvae treatments (*F*_(2, 12)_ = 4.320, *p* = 0.069). In contrast, significant decreases in predation were observed in both fourth instar larvae (*F*_(2, 12)_ = 37.139, *p* < 0.05) and adults (*F*_(2, 12)_ = 17.533, *p* < 0.05) of *H. axyridis* for both insecticide treatments. Specifically, lambda-cyhalothrin and thiamethoxam resulted in reductions of 27.4% and 22.8% in predation number for fourth instar larvae, respectively, while the corresponding reductions for adults were 10.6% and 20.3%.

## 4. Discussion

Lambda-cyhalothrin and thiamethoxam are the primary agents employed in the management of *D. citri*. This study evaluated the toxicity of these insecticides to both *D. citri* and its natural predator, *H. axyridis*. According to the median lethal concentration (LC_50_), both insecticides notably extended the developmental duration of *H. axyridis* and significantly diminished its predation efficiency through two exposure pathways.

Foliar spraying is the predominant method for insecticide application in citrus orchards in Ganzhou. Although insecticides target pests, beneficial arthropods inevitably come into contact with spray residues or droplets while foraging, leading to acute contact toxicity. The median lethal application rate (LR_50_) of thiamethoxam for *H. axyridis* was found to be 0.10 g a.i. ha^−1^ using the glass tube residue method, indicating an unacceptable risk to this predator after exposure both in-field and off-field [23]. Moreover, the oviposition activity of the parasitoid *Aphidius ervi* was significantly reduced at the LD_20_ of lambda-cyhalothrin-treated groups [33], suggesting that lambda-cyhalothrin not only affects predatory natural enemies but also impairs parasitoids. In our study, the LC_50_ values for lambda-cyhalothrin were determined to be 0.870 mg/L for *H. axyridis* and 20.861 mg/L for *D. citri*, while thiamethoxam exhibited LC_50_ values of 2.030 mg/L for *H. axyridis* and 1.658 mg/L for *D. citri*. Lambda-cyhalothrin demonstrated greater toxicity to *H. axyridis*, as indicated by its lower LC_50_ value, suggesting that *H. axyridis* is more susceptible to this insecticide. As a pyrethroid, lambda-cyhalothrin acts as a potent neurotoxic agent that disrupts voltage-sensitive sodium channels, resulting in rapid knockdown upon application [34]. In contrast, thiamethoxam functions as an agonist of nicotinic acetylcholine receptors in the central nervous systems of insects and is an effective second-generation neonicotinoid for controlling *D. citri* [35]. Although thiamethoxam exhibits gastrotoxic, contact, and systemic activities, its contact toxicity may not be as pronounced as that of lambda-cyhalothrin, resulting in lower toxicity towards *H. axyridis* in our insect-dipping method.

Furthermore, even in the absence of direct exposure, predators can experience adverse effects from feeding on insecticide-contaminated prey. Both lambda-cyhalothrin and dimethoate exhibited high toxicity to the larval and adult stages of the two-spot ladybird (*Adalia bipunctata*) when they consumed contaminated green peach aphids (*Myzus persicae*) [36]. In *H. axyridis*, exposure to azadirachtin via treated aphids resulted in an increased antifeedant effect correlated with rising azadirachtin concentrations, alongside alterations in the activities of superoxide dismutase (SOD), peroxidase (POD), and catalase (CAT) enzymes [37]. Nearly all instars of *Coccinella septempunctata* and *H. axyridis* succumbed within three hours of ingesting lambda-cyhalothrin-treated *Acyrthosiphon pisum* [27]. The current study presented similar findings, with the exception of third instar larvae of *H. axyridis*, as second and fourth instars, along with adults, exhibited significantly reduced predatory efficacy when feeding on lambda-cyhalothrin- or thiamethoxam-treated psyllids. Additionally, exposure of *Chrysoperla externa* larvae to plants cultivated from thiamethoxam-treated seeds resulted in sublethal and transgenerational effects, which also influenced *H. axyridis* larvae, leading to decreased pupal survival in the F1 generation [28]. Future research on the impact of insecticide residues in citrus on natural enemies is warranted.

The functional response serves as an effective metric for evaluating the predation efficiency of natural enemies on specific pests in relation to ecological changes [38,39]. The typical type II functional response exhibits a negative density-dependent relationship, characterized by a decline in predation rate at higher prey densities, often illustrated by a hyperbolic curve [40]. This type II response represents the most frequently utilized model of Holling’s classical functional response among natural enemies targeting pests, particularly ladybirds [41]. In our study, we identified the functional response as type II, which remained unchanged following LC_50_ exposure to lambda-cyhalothrin and thiamethoxam, aligning with prior findings concerning *H. axyridis* preying on *D. citri* [13]. Within Holling’s framework, three key parameters, namely instant attack rate (*a*), handling time (*T_h_*), and predation capacity (*a*/*T_h_*), are frequently employed for comparison and analysis. High attack rates coupled with low handling times lead to an increased predation capacity, suggesting greater efficacy as biocontrol agents in the field. Thiamethoxam was shown to impair the predatory ability of *H. axyridis* against *Myzus persicae* in leaf-dip treatments, resulting in decreased attack rates and increased handling times, with an overall reduction in predation by 22.4% [22]. Exposure to azadirachtin-treated aphids also led to diminished instant attack rates and daily maximum predation rates in both *H. axyridis* larvae and adults [37]. Consistent with these findings, our present study demonstrated that after feeding on lambda-cyhalothrin- or thiamethoxam-contaminated psyllids, both the instant attack rate and predation capacity of *H. axyridis* decreased across most developmental stages, particularly in second instar larvae, which exhibited an 85.30% reduction in predation capacity. Conversely, reductions for fourth instar larvae and adults were markedly lower at 7.49% and 6.53%, respectively, indicating that the negative effects on predation efficiency diminish progressively as the predator matures.

As previously discussed, despite extensive research on HLB, an effective therapeutic agent for this troubling disease remains unavailable. Consequently, managing *D. citri* has emerged as the primary strategy for controlling HLB. Implementing biological control methods to lower the density of citrus psyllids could represent a promising approach. A critical requirement for the successful application of natural enemies is the identification of insecticides that are relatively less harmful, facilitating a more effective integration of biological and chemical control strategies. This study revealed that lethal concentrations (LC_50_) of lambda-cyhalothrin and thiamethoxam induce acute toxicity, adversely affecting the biological parameters and functional response of *H. axyridis*. However, further investigation is warranted to assess specific long-term effects, including potential transgenerational impacts. Additionally, in the ingestion exposure treatment, the observed reduction in *H. axyridis* intake may be attributed to an antifeedant effect induced by psyllids contaminated with lambda-cyhalothrin or thiamethoxam. Consequently, targeted behavioral and physiological assays are necessary to accurately identify the specific behavioral traits that are affected.

## 5. Conclusions

In conclusion, we assessed the impact of commonly utilized insecticides, lambda-cyhalothrin and thiamethoxam, on the predation of *D. citri* by *H. axyridis* via two exposure routes. Both insecticides demonstrated acute toxicity at lethal concentrations (LC_50_), negatively affecting the development and predation efficacy of *H. axyridis*. Consequently, the application of lambda-cyhalothrin and thiamethoxam in integrated pest management (IPM) strategies targeting *D. citri* should be approached with caution.

## Figures and Tables

**Table 1:** Toxicity of two pesticides to *H. axyridis* and *D. citri*.

Species	Insecticide	Regression Equation of Toxicity	*n*	*df*	*χ* ^2^	*p*	LC_50_ (mg/L) (95% Confidence Interval)
*H. axyridis*	Lambda-cyhalothrin	y = 0.086 + 1.423x	25	23	4.601	0.999	0.870 (0.651–1.144)
	Thiamethoxam	y = −1.226 + 3.987x	25	23	7.723	0.996	2.030 (1.825–2.260)
*D. citri*	Lambda-cyhalothrin	y = −1.970 + 1.493x	25	23	13.945	0.928	20.861 (17.944–24.322)
	Thiamethoxam	y = −0.302 + 1.458x	25	23	17.074	0.806	1.658 (1.422–1.931)

Note: toxicity regression equation, y in the table represents the probit value, and x represents the log10 of concentration.

**Table 2:** Parameter estimates from Holling’s disc equation for *H. axyridis* subjected to LC_50_ treatments of lambda-cyhalothrin and thiamethoxam.

Stage of *H. axyridis*	Treatment	Functional Response Equation	*χ* ^2^	Instant Attack Rate *a*	Handling Time *T_h_* (d)	Predation Capacity *a*/*T_h_*	Max Predating Rate 1/*T_h_*
Second instar larva	Control	*N*_a_ = 0.9023*N*_0_/(1 + 0.0127*N*_0_)	0.569	0.9023	0.0141	64	70.92
	Lambda-cyhalothrin	*N*_a_ = 0.5079*N*_0_/(1 + 0.0274*N*_0_)	0.108	0.5079	0.0540	9.41	18.52
	Thiamethoxam	*N*_a_ = 0.4972*N*_0_/(1 + 0.0338*N*_0_)	0.312	0.4972	0.0680	7.31	14.71
Third instar larva	Control	*N*_a_ = 0.9728*N*_0_/(1 + 0.0084*N*_0_)	0.356	0.9728	0.0086	113.12	116.28
	Lambda-cyhalothrin	*N*_a_ = 0.8587*N*_0_/(1 + 0.011*N*_0_)	0.177	0.8587	0.0125	68.70	80.00
	Thiamethoxam	*N*_a_ = 0.9042*N*_0_/(1 + 0.012*N*_0_)	0.283	0.8915	0.0140	63.68	71.43
Fourth instar larva	Control	*N*_a_ = 1.0401*N*_0_/(1 + 0.0029*N*_0_)	0.510	1.0401	0.0028	371.46	357.14
	Lambda-cyhalothrin	*N*_a_ = 1.0309*N*_0_/(1 + 0.0031*N*_0_)	0.179	1.0309	0.0030	343.63	333.33
	Thiamethoxam	*N*_a_ = 1.0103*N*_0_/(1 + 0.0029*N*_0_)	0.413	1.0103	0.0029	348.38	344.83
Adult	Control	*N*_a_ = 1.0144*N*_0_/(1 + 0.0029*N*_0_)	0.500	1.0144	0.0029	349.79	344.83
	Lambda-cyhalothrin	*N*_a_ = 1.0135*N*_0_/(1 + 0.0031*N*_0_)	0.777	1.0135	0.0031	326.94	322.58
	Thiamethoxam	*N*_a_ = 1.0378*N*_0_/(1 + 0.0033*N*_0_)	0.314	1.0378	0.0032	324.31	312.50

Note: *N*_a_, number of prey consumed; *N*_0_, initial prey density; *a*, instantaneous attack rate; *T_h_*, handling time; *a*/*T_h_*, predation ability; 1/*T_h_*, maximum predation rate.

**Table 3:** Predation number of contaminated psyllids by *H. axyridis*.

Stage of *H. axyridis*	Density of *D. citri*	Predation Number		
		Control	Lambda-Cyhalothrin	Thiamethoxam
Second instar larva	25	14.67 ± 0.33 a	12.00 ± 1.15 ab	10.00 ± 0.58 b
Third instar larva	60	41.33 ± 1.76 a	41.33 ± 1.45 a	35.33 ± 1.76 a
Fourth instar larva	110	80.33 ± 2.73 a	58.33 ± 1.20 b	62.00 ± 1.53 b
Adult	110	82.00 ± 2.08 a	73.33 ± 2.03 b	65.33 ± 1.85 c

Data in the table are mean ± SE. Means within a row followed by different letters are significantly different (one-way ANOVA followed by Tukey’s HSD test: *p* < 0.05).

## Data Availability

The data presented in this study are available on request from the corresponding authors.

## Supplementary Materials

The following supporting information can be downloaded at: https://www.mdpi.com/article/10.3390/insects16020126/s1, Figure S1: Functional response curves of *Harmonia axyridis* exposed to LC50 concentrations of lambda-cyhalothrin and thiamethoxam while preying on *Diaphorina citri*. The data points indicate the average number of prey consumed, and the curves were generated based on predictions derived from Holling’s disc equation.
