# Supplementary material for: Impact of Direct Contact and Ingestion of Selected Insecticides on the Predator Harmonia axyridis of Citrus Psyllids
Source: Insects. 2025 Jan 27;16(2):126. doi: 10.3390/insects16020126 (PMC11855586; doi:10.3390/insects16020126)
Supplement: Supplementary file 1 [file insects-16-00126-s001.zip › insects-3420300-supplementary.pdf]

Supplementary Material

Impact of Direct Contact and Ingestion of Selected Insecticides on the Predator *Harmonia axyridis* of Citrus Psyllids

Jing Pan [1], Gaoqi Cheng [1], Minjue Liu [1], Xiangfeng Pan [1], Zhigang Ouyang [1,2,3], Zhanjun Lu [1,2,3,*] and Yimin Du [1,2,3,*]

[1] College of Life Sciences, Gannan Normal University, Ganzhou 341000, China; pan215662131@163.com (J.P.)
[2] National Navel Orange Engineering Research Center, Ganzhou 341000, China
[3] Jiangxi Provincial Key Laboratory of Pest and Disease Control of Featured Horticultural Plants, Ganzhou 341000, China
* Correspondence: luzhanjun7@139.com (Z.L.); dym1009@163.com (Y.D.)

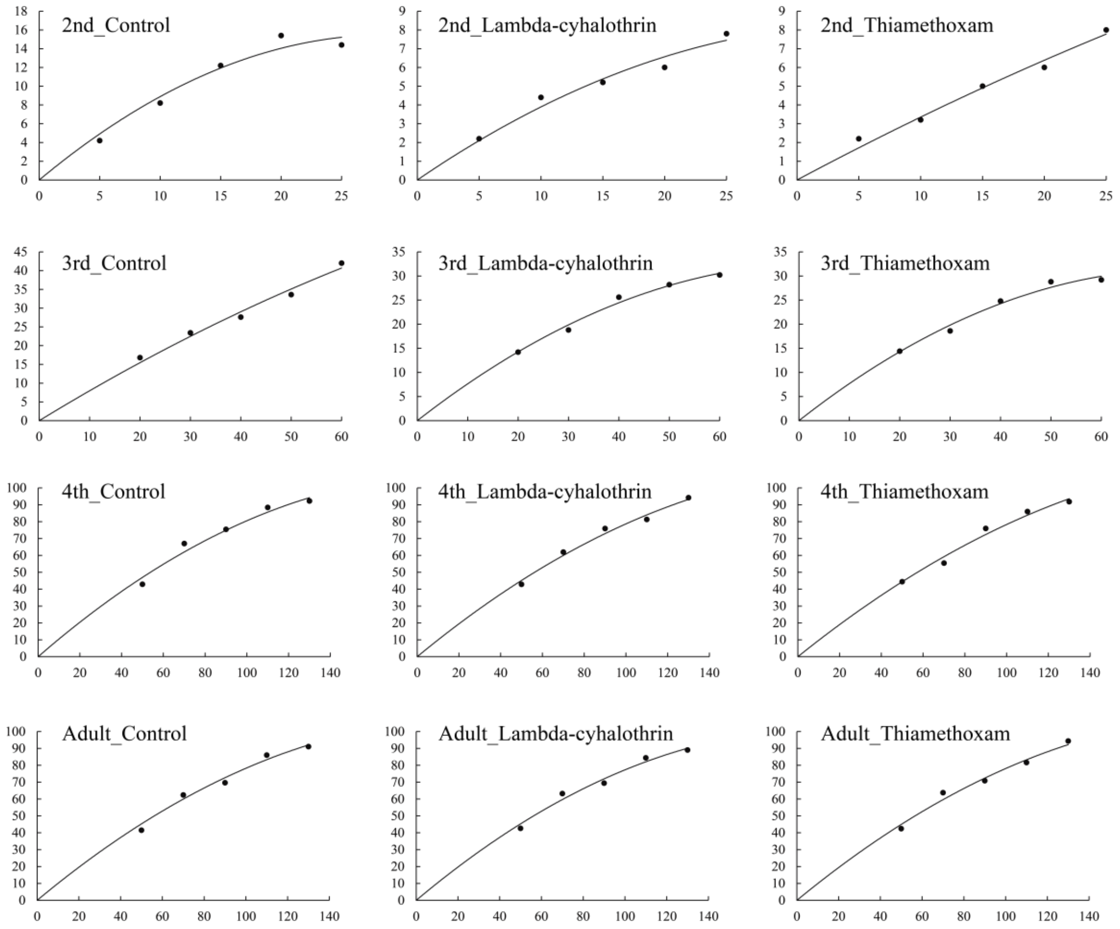

Figure S1. Functional response curves of *Harmonia axyridis* exposed to LC_{50} concentrations of lambda-cyhalothrin and thiamethoxam while preying on *Diaphorina citri*. The data points indicate the average number of prey consumed, and the curves were generated based on predictions derived from Holling's disc equation.
